# Doppler-Based Flow Rate Sensing in Microfluidic Channels

**DOI:** 10.3390/s140916799

**Published:** 2014-09-10

**Authors:** Liron Stern, Avraham Bakal, Mor Tzur, Maya Veinguer, Noa Mazurski, Nadav Cohen, Uriel Levy

**Affiliations:** Department of Applied Physics, The Benin School of Engineering and Computer Science, The Center for Nanoscience and Nanotechnology, The Hebrew University of Jerusalem, Jerusalem 91904, Israel; E-Mails: liron.stern@mail.huji.ac.il (L.S.); avibakal@gmail.com (A.B.); mor.tzur11@gmail.com (M.T.); myview21@yahoo.com (M.V.); noam@savion.huji.ac.il (N.M.); nadav_cohen@mod.gov.il (N.C.)

**Keywords:** micro fluidics devices, sensors, velocimetry

## Abstract

We design, fabricate and experimentally demonstrate a novel generic method to detect flow rates and precise changes of flow velocity in microfluidic devices. Using our method we can measure flow rates of ∼2 mm/s with a resolution of 0.08 mm/s. The operation principle is based on the Doppler shifting of light diffracted from a self-generated periodic array of bubbles within the channel and using self-heterodyne detection to analyze the diffracted light. As such, the device is appealing for variety of “lab on chip” bio-applications where a simple and accurate speed measurement is needed, e.g., for flow-cytometry and cell sorting.

## 1. Introduction

Microfluidics has emerged as a key platform for the miniaturization and automation of “lab on a chip” systems. Nowadays, microfluidic devices are being used in myriad applications, such as chemical analysis and synthesis, sample preparation, mixing, particle sorting, droplet generation, and more [1]. Many of the high throughput applications, such as flow cytometry, particle counting, and particle sorting, require accurate measurement and control of flow rate within the microfluidic channels [2,3]. Thus, a flow sensor is an important component in such systems. Clearly, different types of applications may require specific operation conditions of flow rate sensors. For example, applications, such as high throughput particle counting, require much higher flow rates compared with other applications, such as sample preparation and chemical synthesis. Several types of flow rate sensors were demonstrated over the years, primarily based on thermal, mechanical, electrical, and optical concepts of operation [4–8]. Two prominent approaches for optical velocity measurements are imaging velocimetry and the Laser Doppler veclociemter (LDV). Imaging methods use fast cameras to record the flow and implement different image processing algorithms, such as particle counting, and cross correlations, to analyze the data and obtain an image of the flow rate [9–14].

LDV is based on the well-known fact that light scattered by a moving particle undergoes a Doppler shift. Detection of this shift with extremely high resolution was made possible by heterodyning [15–17] or self-mixing [18–22] the Doppler shifted signal from solid particles introduced or self-occurring (for instance, blood cells [23,24]) in the flow. Due to the sporadic nature of the scattering, such measurements are often limited in their sensitivity, and signal to noise ratios.

To date, there is still a crucial need for miniaturized devices, which can provide high accuracy, high dynamic range and integration flexibility, and at a low cost and ease of operation.

In recent years we are witnessing a rapid development in optofluidics, which essentially represents the effort of bringing together microfluidics and photonics, with the goal of constructing miniaturized fluid based photonic devices and systems. This activity has led to the development of various novel devices, such as optofluidic lasers and tunable optical devices and sensors to name a few. Several review papers describe the activity in this field [25–34].

In this letter we demonstrate a miniaturized optofluidic on-chip flow rate sensor. Our device operates on the basis of measuring the Doppler shift of a periodic array of bubbles generated within a microfluidic channel. In order to measure the Doppler shift, we implement the LDV technique, in the self-heterodyne arrangement. We claim that due to the homogeneity and periodicity of the array of bubbles, high signal to noise ratio velocity measurements are achievable, with high precision and accuracy.

## 2. Experimental Section

**Figure 1.**
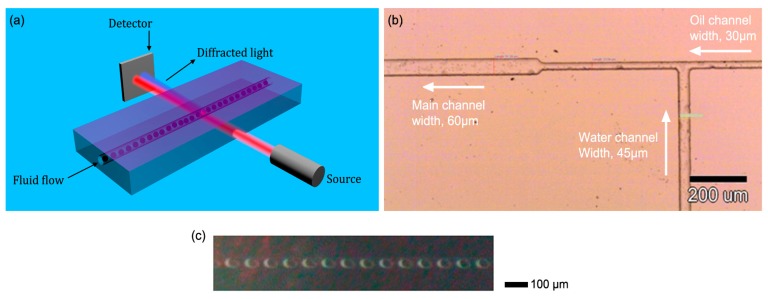
(**a**) Schematic illustration of the optofluidic flow rate sensing device, comprising of microfluidic channels, light source and detector; (**b**) A micrograph showing the fabricated T- junction section in PDMS; (**c**) Photographs showing the periodic bubble array in the channel.

Our proposed approach is described schematically in Figure 1a. A micro-fluidic channel is constructed with a flow of periodically arranged oil bubbles immersed in water acting as a diffraction grating. Owing to the constant flow of bubbles within the channel, the frequency of a diffracted beam will experience a Doppler shift, which is linearly proportional to the flow rate [22,23]. This frequency is measured by heterodyning [16] the zero order beam with the first order beam, resulting in a real time measurement of the flow rate of the channel. We refer to such mixing as self-heterodyning. With this approach, we measured flow rates with a sensitivity in the order of 10 μm/s, limited eventually by the uniformity of the bubble distribution, the uniformity of the flow rate, and by the acquisition time of our system.

A micrograph of our constructed microfluidic device that enables the generation of bubbles within a microfluidic channel is presented in Figure 1b. The device is based on the concept described in references [35–37], where the dimensions and the operation parameters have been optimized to our application. The device was fabricated using standard soft-lithography and is made of poly-dimethyl-siloxane (PDMS).

The fabricated device consists of two inlets and one outlet. One of the inlets is designed for delivering the water (refractive index of n∼1.33) while the other delivers the oil, which has a higher refractive index (n∼1.47). The two channels operate under conditions of continuous flow and are connected via a T-junction, where oil droplets are generated due to the immiscibility of the oil in the water. The widths of the water and oil channels are approximately 45 and 30 μm respectively, whilst their height is ∼10 μm. The dimensions have been chosen in order to enable reasonable flow rates without needing to apply very high-pressure values. In addition, operation at very small heights in the few μm level, and at high aspect ratios (width over height) has a negative effect on the continues creation of bubbles [38] and thus such high aspect ratios should be avoided. The two inlets were connected via Tygon^®^ Tubes to pressure regulators (SMC AW20) which were controlled independently, with typical pressures of up to 2.5 bar. Next, the inlets were filled with distilled water and mineral oil (Sigma-Aldrich). After being connected at the T-junction, the unified channel undergoes a transition from a narrow channel to a wider channel of about 100 μm in order to reduce the resistivity to flow. In Figure 1c a typical micrograph of the oil bubbles is presented. The periodic structure can be clearly observed. Such a mode of operation has been witnessed over the course of a few days, without any evidence of degradation. The period of the bubbles as well as the duty cycle can be tuned by controlling the external pressure of the inlets. Typical pressure is in the order of 1 bar.

Upon illuminating the structure by a normally incident coherent source, light is diffracted into a set of diffraction orders. However, due to the flow, each diffraction order experiences a Doppler shift. The near-field time dependent diffraction pattern is, thus, given by:

(1) $$E(k)\sim \int_{-\infty }^{\infty }\tau (x+tv)e^{\text{ikx}}e^{-\frac{ix^{2}}{z\lambda }}dx=\sum_{n}e^{\frac{i2\pi nv}{\Lambda }t}\int_{-\infty }^{\infty }a_{n}e^{\frac{i2\pi n}{\Lambda }x}e^{\text{ikx}}e^{-\frac{ix^{2}}{z\lambda }}dx$$

where *τ* is the transmission complex amplitude function of the grating, *v* is the translation speed, *z* is the distance between the grating and the screen, *λ* is the wavelength, and *k* is the wave number. Indeed, it can be seen that each Fourier component term is accompanied by a frequency shift of Δ*f* = *nv*/Λ. The spectral spread of this Doppler shift is determined by the uniformity of the diffraction grating, the flow rate, the sampling rate and the acquisition time. In order to measure such frequency shifts, we chose to use a heterodyne approach. In such an arrangement, the frequency shifted beam is combined with a reference unperturbed beam and the beat signal is measured. Here, a more compact and simple approach of self-heterodyning is adopted. Operating in the near field Fresnel regime [39], the various diffraction orders propagate a short distance, and thus overlap. As a result the beam combines both the zero order term, which does not experience Doppler shift, and the higher order, Doppler shifted terms that are described by Equation 1. This is in contrast to the far field regime where the signal degrades due to poor spatial overlap. In order to maximize the beat signal, we choose the height of the channel such that the incident light is diffracted significantly both to the first and the zero diffraction orders. We calculate the zero order and first order efficiencies in and find them to be 0.6 and 0.15, respectively.

## 3. Results and Discussion

### 3.1. Velocity Measurement Calibration Using a Metallic Grating

**Figure 2.**
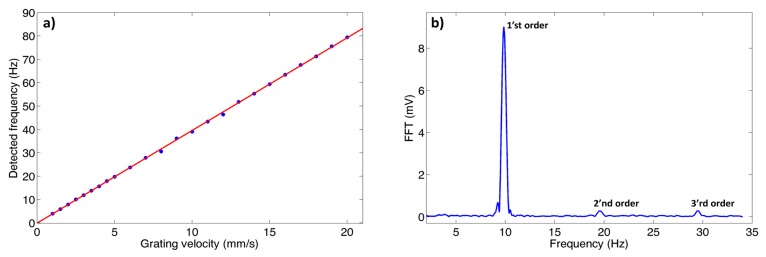
(**a**) Measured frequency shift as a function of the velocity of the grating. The data is based on measuring the first order beat signal; (**b**) Fourier transform of the signal measured by the detector for translation stage speed of 10 mm/s.

Before conducting the actual experiment with the bubble sample, we performed a reference measurement in order to verify and test the feasibility of detecting Doppler shifts created by a moving grating. In order to do so we fabricate a binary amplitude grating with a period of 4 lines/mm. Using a translation stage we shifted the grating at a constant velocity ranging from a few mm/s to 20 mm/s. Next, we illuminated the periodic structure with a collimated HeNe laser beam (1 mm diameter) and collected the light using a conventional silicon based photodetector (TL DET36A). This approach allows to measure the Doppler shift experienced by the diffraction orders of the beam. As mentioned earlier, the different orders overlap spatially at the detector plane, with the beating signal between two adjacent orders yielding a measurable Fourier component corresponding to the frequency difference. We placed the photodetector in the Fresnel near field regime (∼6 cm from the grating's plane, corresponding to a Fresnel number of ∼25) in order to achieve spatial beating between the diffraction orders. In Figure 2a, we plot the measured first order frequency difference as a function of the grating velocity. A linear dependency is clearly observed, with a slope of 4, corresponding to the grating period. In Figure 2b, we plot a typical Fourier transform of the obtained signal. Apart from the DC component three clear Fourier components are visible corresponding to the first three harmonics. The higher harmonics are the result of beating of the higher diffraction order with the zero diffraction order and amongst themselves.

The full width half maximum of the Fourier component is measured to be 0.5 Hz, which is mostly as a result of non-uniformity in the translation speed.

### 3.2. Velocity Measurement of Bubble Array in a Micro Fluidic Channel

**Figure 3.**
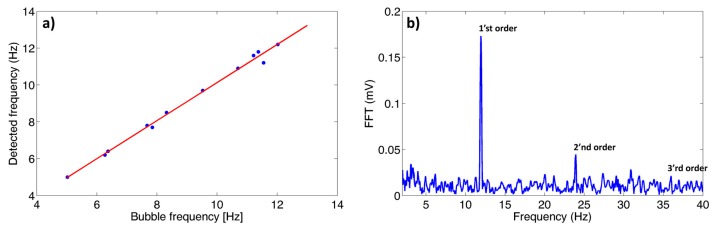
Measured Doppler frequency shift as a function of the bubble arrival rate as measured by a camera. The data is based on measuring the first order beat signal; (**b**) Fourier transform of a typical signal measured by the detector.

Next, we turn into measuring the flow rate in the actual device. As before, we illuminate the sample (this time being the periodic bubble structure) with a collimated HeNe laser beam, in the same heterodyne arrangement mentioned above. In our current demonstration, this Doppler shift corresponds to ∼10 Hz. However, higher rates of operations (*i.e.*, larger velocities or smaller grating periods) are possible yielding higher Doppler shifts, which in principle are easier to measure. In Figure 3a, we plot the measured Doppler frequency as a function of the directly measured bubble frequency, which is estimated by measuring the shift between several consecutive snapshots of a standard video camera. We see an excellent agreement between the Doppler and the direct measurement, where we believe that the small discrepancies between the two are attributed to the accuracy of measuring the bubble speed using a standard camera, rather than the accuracy of the Doppler measurement itself. In Figure 3b, we plot an example of the Fourier transform of the measured signal. Distinct peaks corresponding to the first and second order are evident.

The full width half maximum of the Fourier component is measured to be 0.2 Hz. In this case, the frequency resolution corresponds to 0.05 Hz, which sets a limit on the accuracy of the measurement. We attribute the broadening of this Fourier component to slight non-uniformities in bubble position and local velocity (perhaps due to Brownian motion). In order to convert the Doppler shift measurement to the actual flow velocity, there is a need to extract the period of the bubble grating. Using straightforward techniques of Fourier analysis of photographs similar to that presented in Figure 1c the grating period was found to be 198 μm. With this period we can now translate the Doppler shift to an actual flow velocity of 2.36 mm/s. Assuming that the period is known with great accuracy (a realistic scenario in pre-calibrated devices), the sensitivity of the velocity measurement is inherently limited by the sampling period in the frequency domain (0.05 Hz), resulting in ultimate velocity sensitivity of ∼10 μm/s. Realistically, small uncertainties in grating period and linewidth broadening lead to a slightly lower resolution in measuring the flow velocity. By developing precise mechanisms for measuring the grating period, increasing the sampling rate in the frequency domain and by using advanced algorithms for precise peak estimation, the presented approach should provide high accuracy velocity measurement, in the ∼μm/s range. We note that the described method enables the measurements of flow velocities much higher than reported here. For example, using a fast detector having a bandwidth of ∼1 MHz, flow velocities above 100 m/s could be measured. Not only this capability of measuring such high flow rate is more than sufficient for conventional microfluidic and optofluidic devices, it is even more than sufficient in applications such as measuring the flow rate of jet based dye lasers [40].

## 4. Conclusions

In summary, we demonstrated a flow rate sensor based on measuring the Doppler shift experienced by light, which is diffracted from a microfluidic channel consisting of 1-D period array of oil bubbles. We constructed the device and witnessed stable and controlled periodic bubble flow. Using the device we measured Doppler shifts of ∼10 Hz, corresponding to flow rates of ∼2 mm/s. A good agreement between the predicted and detected Doppler shift was observed.

The device offers two prominent features. First, it introduces a self-generated periodic and homogeneous array of particles. Such an ordered array of bubbles provides a strong and narrow Doppler shift peak in the measured spectrum, corresponding to high SNR and sensitivity. This is as opposed to exploiting random particles in fluids for the measurement of flow rates, an approach, which might be limited by a low SNR as a result of sporadic scattering from the random distribution and the random size of the particles. Furthermore, the technique is all liquid, *i.e.*, both materials are liquids, with no other materials involved. Being an all-liquid approach has the advantage of homogeneity, *i.e.*, the same density of materials all over the channel (in all dimensions) and liquid compatibility.

The concept presented here is very general. It enables to combine any two liquids that do not mix, and have a reasonable contrast in their indices of refraction. Moreover, it is possible to implement such a technique using gas bubbles instead of oil bubbles, in the same manner introduced in reference [37]. However, in some cases, this might not be ideal, as the density of the liquid and the gas is obviously very different.

The proposed approach is expected to provide an integrated, low cost and high performance tool for measuring flow rates over broad range of flow velocities. The demonstrated concept can be further integrated with microfluidic devices in variety of applications such as particle sorting, particle counting, and flow cytometers.

## References

[1] Squires T.M., Quake S.R. (2005). Microfluidics: Fluid physics at the nanoliter scale. Rev. Mod. Phys..

[2] Lien V., Zhao K., Berdichevsky Y., Lo Y.-H. (2005). High-sensitivity cytometric detection using fluidic-photonic integrated circuits with array waveguides. IEEE J. Sel. Top. Quantum Electron..

[3] Godin J., Lien V., Lo Y.-H. (2006). Demonstration of two-dimensional fluidic lens for integration into microfluidic flow cytometers. Appl. Phys. Lett..

[4] Nguyen N.-T., Wereley S.T. (2002). Fundamentals and Applications of Microfluidics.

[5] Ernst H., Jachimowicz A., Urban G.A. (2002). High resolution flow characterization in Bio-MEMS. Sens. Actuators A Phys..

[6] Collins J., Lee A.P. (2004). Microfluidic flow transducer based on the measurement of electrical admittance. Lab Chip.

[7] Lee G.-B., Kuo T.-Y., Wu W.-Y. (2002). A novel micromachined flow sensor using periodic flapping motion of a planar jet impinging on a V-shaped plate. Exp. Therm. Fluid Sci..

[8] Bayraktar T., Pidugu S.B. (2006). Characterization of liquid flows in microfluidic systems. Int. J. Heat Mass Transf..

[9] Santiago J.G., Wereley S.T., Meinhart C.D., Beebe D.J., Adrian R.J. (1998). A particle image velocimetry system for microfluidics. Exp. Fluid..

[10] Cierpka C., Segura R., Hain R., Kähler C.J. (2010). A simple single camera 3C3D velocity measurement technique without errors due to depth of correlation and spatial averaging for microfluidics. Meas. Sci. Technol..

[11] Lindken R., Rossi M., Große S., Westerweel J. (2009). Micro-Particle Image Velocimetry (μPIV): Recent developments, applications, and guidelines. Lab Chip.

[12] Cierpka C., Kähler C.J. (2012). Particle imaging techniques for volumetric three-component (3D3C) velocity measurements in microfluidics. J. Vis..

[13] Wang H.L., Han W., Xu M. The measurements of water flow rates in the straight microchannel based on the scanning micro-PIV technique.

[14] Scarano F. (2013). Tomographic PIV: Principles and practice. Meas. Sci. Technol..

[15] Yeh Y., Cummins H.Z. (1964). Localized fluid flow measurements with an He-Ne Laser spectrometer. Appl. Phys. Lett..

[16] Goldstein R. (1996). Fluid Mechanics Measurements.

[17] Meier A.H., Roesgen T. (2009). Heterodyne Doppler global velocimetry. Exp. Fluid..

[18] Giuliani G., Norgia M., Donati S., Bosch T. (2002). Laser diode self-mixing technique for sensing applications. J. Opt. A: Pure Appl. Opt..

[19] Nikolic M., Lim Y.L., Wilson S.J., Rakic A., Campagnolo L., Perchoux J., Bosch T. Flow profile measurement in micro-channels using changes in laser junction voltage due to self-mixing effect.

[20] Dmitriev A.K., Konovalov A.N., Ul'yanov V.A. (2014). Self-mixing detection of backscattered radiation in a single-mode erbium fibre laser for Doppler spectroscopy and velocity measurements. Quantum Electron..

[21] Alexandrova A.S., Tzoganis V., Welsch C.P. (2014). Self-mixing diode laser interferometry for velocity measurements of different targets. Proc. SPIE.

[22] Nikoli M., Hicks E., Lim Y.L., Bertling K., Raki A.D. (2013). Self-mixing laser Doppler flow sensor: An optofluidic implementation. Appl. Opt..

[23] Stern M.D., Lappe D.L., Bowen P.D., Chimosky J.E., Holloway G.A., Keiser H.R., Bowman R.L. (1977). Continuous measurement of tissue blood flow by laser-Doppler spectroscopy. Am. J. Physiol..

[24] Campagnolo L., Roman S., Perchoux J., Lorthois S. (2012). A new optical feedback interferometer for measuring red blood cell velocity distributions in individual capillaries: A feasibility study in microchannels. Comput. Method. Biomech. Biomed. Eng..

[25] Campbell K., Groisman A., Levy U., Pang L., Mookherjea S., Psaltis D., Fainman Y. (2004). A microfluidic 2 × 2 optical switch. Appl. Phys. Lett..

[26] Erickson D., Rockwood T., Emery T., Scherer A., Psaltis D. (2006). Nanofluidic tuning of photonic crystal circuits. Opt. Lett..

[27] Levy U., Campbell K., Groisman A., Mookherjea S., Fainman Y. (2006). On-chip microfluidic tuning of an optical microring resonator. Appl. Phys. Lett..

[28] Gersborg-Hansen M., Balslev S., Mortensen N.A., Kristensen A. (2005). A Coupled Cavity Micro-fluidic Dye Ring Laser. Microelectron. Eng..

[29] Garstecki P., Fischbach M.A., Whitesides G.M. (2005). Design for mixing using bubbles in branched microfluidic channels. Appl. Phys. Lett..

[30] Li Z., Zhang Z., Scherer A., Psaltis D. (2006). Mechanically tunable optofluidic distributed feedback dye laser. Opt. Express..

[31] Pang L., Chen H.M., Freeman L.M., Fainman Y. (2012). Optofluidic devices and applications in photonics, sensing and imaging. Lab Chip.

[32] Psaltis D., Quake S.R., Yang C. (2006). Developing optofluidic technology through the fusion of microfluidics and optics. Nature.

[33] Monat C., Domachuk P., Eggleton B.J. (2007). Integrated optofluidics: A new river of light. Nat. Photon..

[34] Levy U., Shamai R. (2008). Tunable optofluidic devices. Microfluid. Nanofluid..

[35] Huebner A., Sharma S., Srisa-Art M., Hollfelder F., Edel J.B., Demello A.J. (2008). Microdroplets: A sea of applications?. Lab Chip.

[36] Beatus T., Bar-Ziv R., Tlusty T. (2007). Anomalous Microfluidic Phonons Induced by the Interplay of Hydrodynamic Screening and Incompressibility. Phys. Rev. Lett..

[37] Garstecki P., Fuerstman M.J., Stone H.A., Whitesides G.M. (2006). Formation of droplets and bubbles in a microfluidic T-junction—scaling and mechanism of break-up. Lab Chip.

[38] Beatus T., Bar-Ziv R.H., Tlusty T. (2012). The physics of 2D microfluidic droplet ensembles. Phys. Rep..

[39] Goodman J. (2004). Introduction to Fourier Optics.

[40] Wellegehausen B., Welling H., Beigang R. (1974). A narrowband jet stream dye laser. Appl. Phys..

